# COVID-19 knowledge, attitudes and practices in Thabo Mofutsanyana District, South Africa, 2022

**DOI:** 10.4102/jphia.v16i1.885

**Published:** 2025-06-19

**Authors:** Inge Kleinhans, Siphesihle K. Mahanjana, Lehlohonolo Kumalo, Brian Brümmer, Ashley Chitaka, Zandile D. Nukeri, Fiona Els, Sizeka Mashele, Michelle Groome, Natalie Mayet, Ramasedi S. Mokoena, Emily B. Atuheire, Joy I. Ebonwu

**Affiliations:** 1 Division of Public Health Surveillance and Response, National Institute for Communicable Diseases, National Health Laboratory Service, Johannesburg, South Africa; 2 Department of Community Health, Faculty of Health Sciences, Sefako Makgatho Health Sciences University, Pretoria, South Africa; 3 Centre for Vaccines and Immunology, National Institute for Communicable Diseases, Division of the National Health Laboratory Service, Johannesburg, South Africa; 4 Gauteng City-Region Observatory, Johannesburg, South Africa; 5 National Cancer Registry, National Institute for Communicable Diseases, National Health Laboratory Service, Johannesburg, South Africa; 6 Society, Gender and Health Unit, Faculty of Epidemiology and Public Health, Swiss Tropical and Public Health Institute, Allschwill, Switzerland; 7 Faculty of Medicine, University of Basel, Basel, Switzerland; 8 Vaccines and Infectious Diseases Analytics Research Unit, Faculty of Health Sciences, University of the Witwatersrand, Johannesburg, South Africa; 9 National Institute for Communicable Diseases, National Health Laboratory Service, Johannesburg, South Africa; 10 Department of Community Health, Faculty of Health Sciences, University of the Free State, Bloemfontein, South Africa; 11 Division of Surveillance and Disease Intelligence, Africa Centres for Disease Control and Prevention, Addis Ababa, Ethiopia

**Keywords:** COVID-19, knowledge, attitudes, practices, community deaths, Thabo Mofutsanyana, Free State, South Africa

## Abstract

**Background:**

Adherence to COVID-19 prevention and control measures is related to people’s knowledge, attitudes and practices.

**Setting:**

In Thabo Mofutsanyana District, the proportion of reported community COVID-19-related deaths was higher than in-facility reported deaths.

**Aim:**

To assess knowledge, attitudes and practices of the community towards COVID-19.

**Methods:**

A survey was conducted among consenting adults from 28 February 2022 to 4 March 2022. An interviewer-administered questionnaire was used for data collection. Descriptive statistics was used to describe the responses and logistic regression used to assess factors associated with poor knowledge towards COVID-19.

**Results:**

A total of 551 participants’ data were analysed, most of whom were < 40 years (63%) and female (68%). Despite 43.4% having education levels below high school, 89% knew that anyone could contract COVID-19, mainly through television and/or radio (74%) and social media (53%). The majority practiced mask-wearing (84%) and social distancing (80%), while 65% indicated they will use home remedies if there was severe COVID-19 infection. Older age group (OR = 2.40; 95% CI 1.17-4.89; *p* = 0.015), higher education level (OR = 0.59; 95% CI 0.39–0.87; *p* = 0.009) and higher monthly income were each associated with poor knowledge towards COVID-19 but the significance did not remain in multivariate model.

**Conclusion:**

Participants had good knowledge of COVID-19; however, a high proportion supported the use of home remedies in severe COVID-19 infections. This underscores the need to enhance the health-seeking behaviour of communities through health education and community engagement, using television and/or radio and social media.

**Contribution:**

Study findings are useful to inform preparedness and response strategies in communities.

## Introduction

COVID-19 caused by the novel severe acute respiratory syndrome coronavirus 2 (SARS-CoV-2), was first reported in Wuhan city, Hubei Province of China on 31 December 2019 and declared a Public Health Emergency of International Concern (PHEIC) by the World Health Organization (WHO).^1^ As of 31 December 2022, more than 655 million COVID-19 cases and over 6 million deaths were recorded globally.^2^ South Africa experienced five waves of the pandemic, with about 4 million cases and 102 568 deaths reported as of 31 December 2022.^3^ Following the notification of the first imported COVID-19 case in South Africa on 05 March 2020, the country implemented response and control measures to fight the pandemic. The district COVID-19 response priorities were in line with the national priorities, focusing on overcoming the struggles of improving capacity for emergency preparedness and response activities related to risk communication and community engagement, epidemiology and active surveillance, laboratory testing, case management (including infection prevention and control) and vaccination.^4^

The knowledge, attitudes and practice (KAP) of communities of a disease play a critical role in disease control.^5,6^ Several COVID-19 KAP surveys conducted in other countries provide evidence that knowledge plays an important role in the attitude and behaviours commonly observed in the communities during this pandemic.^7,8,9,10^ Multiple factors contribute toward behaviours that either promote or mitigate the spread of the disease. Misinformation about COVID-19 and poor attitudes may lead to increased fear and panic in the community,^11,12^ resulting in a delay in health-seeking behaviour despite severe disease.

Thabo Mofutsanyana District, Free State province, South Africa, experienced high SARS-CoV-2 transmission, with 40 758 cases and 2031 deaths reported as of 11 April 2022. Active COVID-19 mortality surveillance in the district revealed a high proportion of community deaths (56%; *n* = 1135/2031)^5^ compared to in-facility deaths. It is not clear why most of the deaths occurred in the community; however, it has been postulated that myths associated with COVID-19 hospitalisation of severe cases and vaccination may have been driving the community deaths. These perceptions may be influenced by the peoples’ knowledge, attitudes and practices towards COVID-19 in general. To gain a better understanding, and to improve the control measures and target interventions to the identified priority gaps, a KAP study was conducted. This study aimed to assess the community’s knowledge, attitude and practices towards COVID-19 in Thabo Mofutsanyana District. Specifically, our study was structured to describe the socio-demographic characteristics of the study participants, discuss their KAP towards COVID-19 and determine the socio-demographic factors associated with participants’ level of knowledge on COVID-19. The study findings may provide a better understanding of community-related factors associated with COVID-19 infection and deaths and help inform decisions and priority focus for COVID-19 response and future pandemics in the district.

## Research methods and design

### Study design, setting and population

A field-based cross-sectional survey was conducted in Thabo Mofutsanyana District, from 28 February 2022 to 04 March 2022. Thabo Mofutsanyana is one of the five districts in the Free State province and has an estimated 2020 mid-year population of 764 285. In Thabo Mofutsanyana District, the proportion of reported community COVID-19-related deaths was higher than the in-facility reported deaths. It comprises six sub-districts, namely Maluti-A-Phofung, Dihlabeng, Setsoto, Nketoana, Phumelela and Mantsopa. Study participation was open to any consenting adult community member (≥ 18 years), willing to complete interviewer-administered questionnaires and not confirmed or suspected to have COVID-19 at the time of the interview.

### Sample size and sampling

Convenient sampling technique was used for the cross-sectional survey because of the inappropriateness of using probability sampling technique during the period of the pandemic. A total of 695 participants were enrolled in the study. The sample size was determined using the single population proportion formula:


n=Z2p (1−p)/d2 [Eqn 1]

In the absence of estimates for the level of knowledge, attitudes and perceptions of study participants regarding COVID-19, an expected prevalence of 50% of participants having adequate knowledge of this subject was applied. A 95% confidence interval (CI) with a 5% margin of error was used. This yielded a minimum sample size of 384. Furthermore, a 4% non-response rate was applied which increased the sample size to 400. This sample was proportionally allocated to the six sub-districts based on the number of deaths between March 2020 and August 2021. The sample size for each district was determined as follows: proportion of total death in each sub-district (A) was calculated as total deaths in a sub-district divided by the total deaths in all six sub-districts, multiplied by 100. The minimum sample size for each sub-district was then calculated as the product of the proportion of total death in each sub-district and target minimum sample size (*n* = 400). Consecutive community members were recruited in each sub-district until the required minimum sample size was met.

### Data collection, study variables and scoring

Questionnaire used for data collection was compiled and adapted from previous COVID-19 KAP studies.^5,6,7,8,9,10,13^ The questionnaire was shared with the study team for content validity and piloted among volunteers, who were not part of the survey, to ensure appropriateness and linguistic understanding. Trained study personnel used structured interviewer-administered questionnaires for data collection, in the appropriate local language. The questionnaire consisted of socio-demographic characteristics including age, sex, marital status, education level, employment status, monthly household income and household size, as well as KAP of the community towards COVID-19. The response of the participants to the KAP questions was scored and categorised using modified Bloom’s cut-off points (80% – 100%, 60% – 79% and < 60%) from previous studies.^7,14^

The participant’s knowledge about COVID-19 was assessed using eight questions that covered transmission, symptoms, prevention and outcome. A value of 1 was assigned for every correctly chosen response and 0 for incorrect and/or unsure response. The remaining two questions required multiple responses and assigned values were based on the correct responses. The multiple-choice questions had a cumulative score range of 0 to 8 points for each participant. Hence, the aggregate score for all eight questions assessing knowledge ranged from 0 to 14 points for each participant. For each participant, knowledge scores were calculated as a proportion of correctly answered questions. The participants’ overall knowledge was categorised as good if the score was greater or equal to 80% (12 or above), moderate if the score was between 60% and 79% (9 to 11) and poor if the score was less than 60% (< 9).

Attitude towards COVID-19 was assessed using seven five-point Likert scale questions and the responses were graded as: 1 for ‘strongly disagree’, 2 for ‘disagree’, 3 for ‘neutral’, 4 for ‘agree’ and 5 for ‘strongly agree’. However, the response scores were reversed for the question that addressed the use of home remedies as the best way to avoid death because of severe COVID-19 infection: 5 for ‘strongly disagree’, 4 for ‘disagree’, 3 for ‘neutral’, 2 for ‘agree’ and 1 for ‘strongly agree’. The aggregate score for all seven questions ranged from 7 to 35 for each participant. The overall attitude was categorised as good if the score was greater or equal to 80% (28 and above), moderate if the score was between 60% and 79% (21 to 27) and poor if the score was less than 60% (< 21).

Three questions were used to assess participants’ practices towards COVID-19, covering precautionary measures against COVID-19 and willingness to test for and vaccinate against COVID-19. The precautionary measure question had six multiple responses and a score of 1 was assigned for every correct response. For the remaining two questions, a score of 1 was assigned if there was a willing response and a score of 0 for unwilling or ‘don’t know’ responses. The cumulative score for the three questions ranged from 0 to 8 for each participant and scores ≥ 80% (7 to 8) were used to determine good practice. The cut-off score for moderate practice was 60% – 79% (5 to 6) and < 60% (≤ 4) for poor practice.

### Data analysis

Data from Microsoft Google forms were managed in Microsoft^®^ Excel 2016 and exported to Stata version 18 (StataCorp LP, College Station, Texas, United States [US]) for analysis. Categorical data were summarised as frequencies and proportions. Univariate and multivariate logistic regression analyses were used to determine the socio-demographic factors associated with participants’ level of knowledge on COVID-19. Variables with *p* < 0.2 in univariate analysis were entered into the multivariate model. A *p* < 0.05 in the multivariate analysis was considered statistically significant and 95% CI were used to estimate precision.

### Ethical considerations

Ethical clearance to conduct this study was obtained from the University of the Free State Faculty of Health Sciences Research Ethics Committee (No. UFS-HSD2021/1828/2903), and permissions were granted by the Free State Department of Health and Thabo Mofutsanyana District. Each participant provided verbal informed consent prior to data collection.

## Results

### Socio-demographic characteristics

Of the 695 participants enrolled in the study, 551 were included in the analysis after deduplication and removal of incomplete responses. Most participants who provided responses were females (68%; *n* = 375/551), single (63.5%; *n* = 350/551), less than 40 years (62.8%; *n* = 346/551), unemployed (65.2%; *n* = 359/551), from Maluti-A-Phofung sub-district (50.6%; *n* = 279/551) and had a monthly household income of less than R2000.00 (61.2%; *n* = 337/551). Furthermore, 97.8% (*n* = 539/551) of participants reported living with 2 or more people in their household. With regards to the level of education, 56.6% (*n* = 312/551) reported having attained high school and higher. The socio-demographic characteristics of the participants are summarised in Table 1.

**TABLE 1:** Socio-demographic characteristics of the participants, Thabo Mofutsanyana District, 2022.

Characteristics	Frequency (*n*)	%
**Sex**		
Female	375	68.0
Male	176	32.0
**Age category (years)**		
18–29	152	28.0
30–39	194	35.0
40–49	106	19.0
50–59	54	10.0
60+	45	8.0
**Education level**		
None	14	2.5
Primary	50	9.1
Secondary but less than high school (Matric)	175	31.8
Completed high school	203	36.8
Tertiary	109	19.8
**Monthly household income**		
R0.00	173	31.4
R1.00 – R1999.00	164	29.8
R2000.00 – R3999.00	108	19.6
R4000.00 – R9999.00	65	11.8
R10 000.00 – R29 999.00	35	6.4
> R30 000.00	6	1.1
**Marital status**		
Single	350	63.5
Married	126	22.9
Divorced	10	1.8
Widowed	28	5.1
Living with a partner	37	6.7
**Employed**		
No	359	65.2
Yes	192	34.9
**Number of people in household**		
1	12	2.2
2	113	20.5
3–4	322	58.4
5+	104	18.9
**Sub-district**		
Dihlabeng	123	22.3
Maluti A Pofung	279	50.6
Mantsopa	17	3.1
Nketoana	37	6.7
Phumelela	30	5.4
Setsoto	65	11.8

### Knowledge towards COVID-19

**FIGURE 1:**
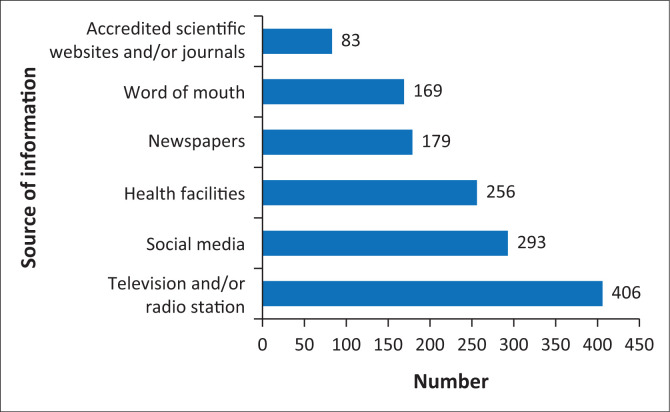
Source of information about COVID-19 in Thabo Mofutsanyane District, 2022.

The majority of participants reported television and/or radio stations (74%; *n* = 406/549) and social media (53.4%; *n* = 293/549) as their sources of COVID-19 health information (Figure 1). Regarding transmission, 88.6% (*n* = 483/545) of the participants agreed that anyone could contract COVID-19. Touching contaminated surfaces and respiratory droplets of infected individuals were reported as main modes of COVID-19 transmission by 56.8% (*n* = 306/539) and 35.8% (*n* = 193/539) participants, respectively. Most of the participants reported that chills (29.9%; *n* = 163/545), diarrhoea (15.4%; *n* = 84/545), body aches (12.3%; *n* = 67/545), as well as coughing (9.7%; *n* = 53/545) were common symptoms of the disease. Most participants (69%; *n* = 376/545) knew the recommended time to test for COVID-19 and 76.3% (*n* = 409/536) agreed that one could be infected with COVID-19 and be asymptomatic (Table 2).

**TABLE 2:** Knowledge of the participants towards COVID-19 in Thabo Mofutsanyane District, 2022.

Variables	Frequency (*n*)	%
**Who can get COVID-19? (*n* = 545)**		
Anyone	483	88.6
Children	1	0.2
Different types of cultures	14	2.6
People with other diseases	15	2.8
The elderly	32	5.9
**Please select the most appropriate definition of a ‘close contact’? (*n* = 530)**		
Going to a shop where a person with COVID-19 has been	139	26.2
In a confined space for more than 15 min without any mask	348	65.7
Talking briefly to a person while both are wearing a mask	38	7.2
Don’t know	5	0.9
**When should you test for COVID-19? (*n* = 545)**		
Every week	26	4.8
If I am a close contact	136	25.0
If I have symptoms of COVID-19	376	69.0
I don’t need to test to know if I have COVID-19	7	1.3
**Not everyone who gets infected with COVID-19 will be severely affected, but it is possible to die from COVID-19 if you are severely affected (*n* = 536)**		
False	74	13.8
True	409	76.3
Unsure	53	9.9
**It is possible to be infected with COVID-19 and still not have any symptoms (*n* = 542)**		
False	58	10.7
True	443	81.7
Unsure	41	7.6
**What are the common symptoms of COVID-19? (multiple answer, *n* = 545)**		
Cough	53	9.7
Shortness of breath	39	7.2
Loss of taste and/or smell	46	8.4
Fever	44	8.1
Runny nose	49	9.0
Body aches	67	12.3
Diarrhoea	84	15.4
Chills	163	29.9
**How is COVID-19 spread? (multiple answer, *n* = 539)**		
Droplets	193	35.8
Touching surfaces	306	56.8
Cellular network 5G	33	6.1
It cannot spread	7	1.3

### Attitude towards COVID-19

**FIGURE 2:**
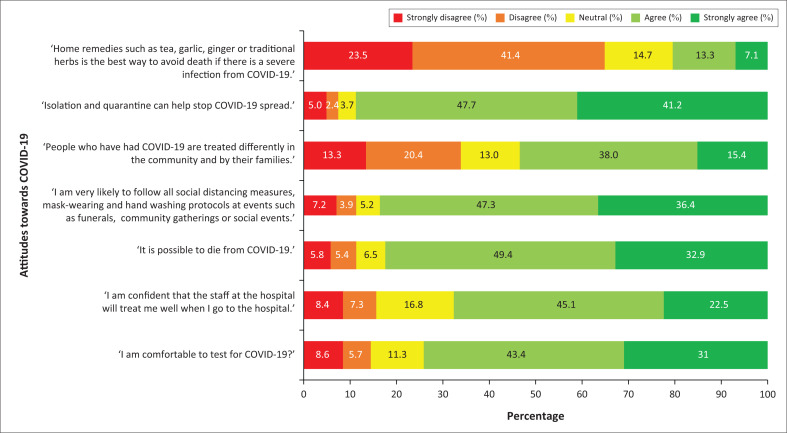
Attitudes towards COVID-19 among participants in Thabo Mofutsanyane District, 2022.

Of the 551 respondents, majority agreed that they are comfortable to test for COVID-19 (74.5%; *n* = 408/548), confident of being treated well at a healthcare facility (66.2%; *n* = 363/548) and would adhere to non-pharmaceutical interventions (83.7%; *n* = 453/541). They also agreed that there was a stigma associated with COVID-19 (53.3%; *n* = 288/540) and that isolation and quarantine are effective at reducing transmission (88.9%; *n* = 479/539). However, most (64.9%; *n* = 348/536) of the participants indicated that the use of home remedies could avoid severe COVID-19 deaths (Figure 2).

### Practices towards COVID-19

Wearing of masks (84.2%; *n* = 458/544), social distancing (80.0%; *n* = 435/544) and sanitising with alcohol (72.6%; *n* = 395/544) were the most recorded non-pharmaceutical prevention practices. Although over half of the participants (57.7%; *n* = 311/539) reported they had not experienced any COVID-19 symptoms since the start of the pandemic, most (82.0%; *n* = 442/539) indicated they would test for COVID-19 if symptomatic and 90.6% (*n* = 490/541) were willing to get vaccinated against COVID-19. With regards to access to vaccination sites, 80.6% (*n* = 435/544) participants reported they had one dose within their community while 3.7% (*n* = 20/544) indicated the vaccination sites were 20 km away from them and 4.1% (*n* = 22/544) did not know where the sites were (Table 3).

**TABLE 3a:** Practices towards COVID-19 in Thabo Mofutsanyane District, 2022.

Characteristics	Frequency (*n*)	%
**Access to vaccination site (*n* = 544)**		
In my community	435	80.6
It is more than 10 km away	63	11.7
It is more than 20 km away	20	3.7
I don’t know	22	4.1
**Prevention of COVID-19 practices (*n* = 544)**		
Mask-wearing	458	84.2
Social distancing	435	80.0
Sanitising with alcohol	395	72.6
Hand washing with soap and water	390	71.7
Avoiding crowded spaces	328	60.3
Ensuring fresh air flow	216	39.7

**TABLE 3b:** Practices towards COVID-19 in Thabo Mofutsanyane District, 2022.

Yes or No practice questions	Yes		No		I don’t know	
	*n*	%	*n*	%	*n*	%
Have you experienced any symptoms of COVID-19 such as coughing, shortness of breath, loss of taste or smell since the start of the pandemic? (*n* = 539)	212	39.3	311	57.7	16	2.9
Would you test for COVID-19 if experiencing symptoms? (*n* = 539)	442	82.0	87	16.1	10	1.8
Would you vaccinate against COVID-19? (*n* = 541)	490	90.6	51	9.4	-	-

### Knowledge, attitudes and practice scores and logistical regression

Overall, 23% of the participants had poor knowledge towards COVID-19 and the attitude of 58% of the participants was classified as moderate. Only 44% of the study participants had good COVID-19 practices (Table 4).

**TABLE 4:** Knowledge, attitudes and practice scores for Thabo Mofutsanyana District, 2022 (*N* = 551).

KAP scores	Knowledge		Attitudes		Practices	
	*n*	%	*n*	%	*n*	%
Poor	127	23	94	94	141	26
Moderate	146	7	320	320	166	30
Good	278	51	137	137	244	44

KAP, knowledge, attitude, practices.

Table 5 shows the factors associated with poor knowledge towards COVID-19. At univariate analysis, being older than 60 years associated with a higher odds of poor knowledge towards COVID-19 compared to those below 30 years (odds ratio [OR] = 2.40; 95% CI: 1.17–4.89; *p* = 0.015). Those that have attained high school education and higher (OR = 0.59; 95% CI: 0.39–0.87; *p* = 0.009) and those with higher monthly income had lower odds of poor knowledge. However, all the observed univariate associations did not retain significance at multivariate analysis.

**TABLE 5:** Factors associated with poor knowledge towards COVID-19 in Thabo Mofutsanyana District, 2022.

Characteristics	Knowledge				Univariate analysis			Multivariable analysis		
	Good		Poor		OR	95% CI	*p*	aOR	95% CI	*p*
	*n*	%	*n*	%						
**Age category (years)**	424	77.0	127	23.0	-	-	-	-	-	-
18–29	119	28.0	33	26.0	-	-	-	-	-	-
30–39	156	37.0	38	30.0	0.88	0.52–1.49	0.628	0.91	0.53–1.57	0.738
40–49	80	19.0	26	20.0	1.17	0.65–2.10	0.596	1.23	0.64–2.33	0.533
50–59	42	9.9	12	9.4	1.03	0.47–2.14	0.938	0.95	0.39–2.22	0.904
60+	27	6.4	18	14.0	2.40	1.17–4.89	0.015	2.05	0.83–5.00	0.116
**Sex**										
Female	285	67.0	90	71.0	-	-	-	-	-	-
Male	139	33.0	37	29.0	0.84	0.54–1.29	0.439	-	-	-
**Monthly household income**										
R0.00	130	31.0	43	34.0	-	-	-	-	-	-
R1.00 – R1999.00	121	29.0	43	34.0	1.07	0.66–1.76	0.774	0.90	0.53–1.52	0.701
R2000.00 – R3999.00	79	19.0	29	23.0	1.11	0.64–1.91	0.709	1.17	0.65–2.10	0.589
R4000.00 – R9999.00	57	13.0	8	6.3	0.42	0.18–0.92	0.040	0.45	0.18–1.01	0.065
R10 000.00 – R29 999.00	31	7.3	4	3.1	0.39	0.11–1.06	0.093	0.43	0.12–1.23	0.148
> R30 000.00	6	1.4	0	0.0	0.00	-	0.981	0.00	-	0.981
**Marital status**										
Single	272	64.0	78	61.0	-	-	-	-	-	-
Married	98	23.0	28	22.0	1.00	0.60–1.61	0.988	0.87	0.48–1.52	0.622
Divorced	9	2.1	1	0.8	0.39	0.02–2.11	0.372	0.35	0.02–2.19	0.343
Widowed	16	3.8	12	9.4	2.62	1.16–5.74	0.017	1.72	0.66–4.35	0.256
Living with a partner	29	6.8	8	6.3	0.96	0.40–2.10	0.926	0.88	0.36–1.98	0.776
**Employment status**	154	36.0	38	30.0	-	-	-	-	-	-
No	-	-	-	-	-	-	-	-	-	-
Yes	-	-	-	-	0.75	0.48–1.14	0.185	-	-	-
**Education level**										
Lower than high school	171	40.0	68	54.0	-	-	-	-	-	-
High school and higher	253	60.0	59	46.0	0.59	0.39–0.87	0.009	0.76	0.48–1.21	0.246

OR, odds ratio; aOR, adjusted odds ration; CI, confidence interval.

## Discussion

Although public health interventions were implemented at national, provincial and district levels in South Africa, enhanced community mortality surveillance in Thabo Mofutsanyane (TM) District in the Free State revealed a high proportion of COVID-19-related community deaths.^4^ The majority of respondents were female and less than 40 years, in line with previous studies conducted in Uganda,^5^ Malawi,^8^ Ethiopia,^9^ India^15^ and South Africa.^16^ Most of the participants had good knowledge (good and moderate combined) about COVID-19. This aligns with a global systematic review where knowledge scores ranged from 72% for low-income countries to 79% for upper middle-income countries.^17^

The main sources of COVID-19 health information were television and/or radio stations and social media. This is similar to KAP studies conducted in Uganda, Ethiopia, Nigeria and Egypt.^5,7,9,18,19^ Although the government provided health information using these platforms, the wide access to social media introduces the risk of infodemics, which the authorities should mitigate by proper risk communication.^20^ Most of the participants knew the common symptoms of COVID-19. The efforts made by the government in disseminating health information, including health campaigns, may have contributed to this knowledge.

Our study observed an association between lower education level and poor knowledge of COVID-19, in line with other published reports from the Middle East and Africa.^6,7,8,9,13,19,21^ Typically, this association has been explained by the enhanced capacity to understand and accurately interpret health information among individuals with a higher educational background.^17^ Furthermore, we found that the higher the income level, the better the knowledge towards COVID-19. Numerous studies conducted in Ethiopia, Nigeria, Egypt, and Saudi Arabia documented similar observations.^9,18,19,21^ This highlights how financial resources facilitate access to educational materials, whether online or physical, thereby enhancing knowledge across diverse subjects. The significance of employment and income in relation to knowledge has been well-established.^9,18,19,21^ This suggests that health education needs to reach individuals from a low socio-economic status and be tailored to their specific needs and level of understanding.

Attitudes and practices are influenced by knowledge.^21^ The knowledge scores found in this study underscore the consequent moderate attitude among participants. Greater efforts to improve knowledge are required to encourage more positive attitudes, which can ultimately translate into good practices. Of note is the large proportion of participants (64.9%) who believed that home remedies, such as tea, garlic, ginger or traditional herbs were the best way to avoid severe disease and death if there was a severe infection from COVID-19. Studies done in other countries such as Ethiopia, Malawi and India identified similar results among participants.^8,9,22^ The findings in this study may indicate a lack of trust in conventional biomedical medicine for severe cases of COVID-19 leading participants to favour traditional methods and remedies which they were more familiar with. The exact reasons for these attitudes require further research. Whether historically associated with the government’s stance on antiretroviral therapy during the early 2000’s^23^ or a function of high levels of misinformation during the COVID-19 pandemic^24^ is an interesting area for inquiry.

A review of KAP studies in sub-Saharan Africa revealed mixed results, where even though knowledge was adequate, practices were often inadequate.^25^ The majority of participants in this study practiced mask-wearing (84%), social distancing (80%) as well as sanitising with alcohol (73%). This was in contrast to a study done in Uganda where only 16% of the participants practiced mask-wearing because of the scarcity and unaffordability of face masks.^5^ The South African government successfully addressed this challenge by enforcing mask-wearing regulations and also permitting individuals to utilise any cloth material as a mask, resulting in a surge in the number of people wearing face masks.^26^ Somewhat reassuringly, 91% of participants indicated that they would vaccinate against COVID-19.

Our study had some limitations. It is possible that respondents gave socially desirable responses to the questions. In addition, the study was conducted in only one district in the Free State province, with a unique socio-demographic context, thereby limiting generalisability. Moreover, as a cross-sectional study, temporal association between observations and outcomes is unable to be demonstrated, limiting causal inference. Other attributable factors for cause of death for example poor nutritional status, excessive alcohol use, environmental exposure etc. will require further investigation.

Risk communication and community engagement (RCCE) is a critical component in outbreak response, especially when it comes to social and behavioural changes needed for the uptake of health interventions. It plays a crucial role in disseminating information, building trust and addressing misconceptions,^27^ which were gaps identified in this study regarding the pandemic response. In addition, RCCE has a great impact on strengthening health systems, which are usually disrupted during outbreaks. Although the COVID-19 pandemic has been controlled and the country is no longer in a national state of disaster, findings from this study provide invaluable insights for pandemic preparedness and response. Understanding the community’s KAP towards the COVID-19 pandemic gives authorities and decision-makers a greater awareness of the necessary interventions to implement to ensure the highest possible uptake of infection prevention and control practices for possible future pandemics.

## Conclusion

This study highlights the need for strengthened pandemic preparedness and response including targeted RCCE in the country using data-driven decision making. Through RCCE, communities are kept well-informed and any needs that arise during emergency responses can be adequately managed. Overall, the community had good KAP; however, the relatively high proportion of people supporting the use of home remedies for severe COVID-19 infections is concerning. This is an area that requires further research particularly when it applies to the use of traditional medication. The health-seeking behaviour of communities can be enhanced through the use of educational materials on television, radio, social media etc., as most people access these platforms.
